# Second-hand Smoke Exposure Among Home Care Workers (HCWs) in Scotland

**DOI:** 10.1093/annweh/wxac066

**Published:** 2022-10-03

**Authors:** Ruaraidh Dobson, Rachel O’Donnell, Mary McGibbon, Sean Semple

**Affiliations:** Institute for Social Marketing, Pathfoor Building, University of Stirling, Stirling, FK9 4LA, UK; Institute for Social Marketing, Pathfoor Building, University of Stirling, Stirling, FK9 4LA, UK; NHS Lanarkshire, Beckford Street, Hamilton, ML3 0TA, UK; Institute for Social Marketing, Pathfoor Building, University of Stirling, Stirling, FK9 4LA, UK

**Keywords:** exposure assessment, home care workers, second-hand tobacco smoke

## Abstract

**Objectives:**

Second-hand tobacco smoke (SHS) is a serious cause of ill-health, and concern around SHS exposure at work has driven legislation in public places. In Scotland, most workers are now protected from SHS at work. However, home care workers (HCWs) may still be exposed, as they enter private homes where smoking is unregulated. In this study, we aimed to understand the extent, duration and intensity of that exposure among HCWs in Lanarkshire, Scotland.

**Methods:**

We surveyed HCWs in four organisations involved in providing care at home: a public healthcare agency (NHS Lanarkshire), two local government entities and a private healthcare company. We also conducted personal exposure monitoring (PEM) of exposure to airborne nicotine and SHS-related fine particulate matter (PM_2.5_) with 32 HCWs.

**Results:**

The vast majority of HCWs surveyed reported being exposed to SHS at work (395/537, 74%), and 50% of those who reported exposure in the home indicated daily exposure. We conducted PEM over 82 home visits, with 21% (17) demonstrating PM_2.5_ concentrations in excess of the WHO’s 2010 air quality guideline limit for 24 h exposure. Duration of exposure to SHS tended to be short and as a result all nicotine samples were below the limit of quantification.

**Conclusions:**

Most HCWs are exposed to minimal levels of SHS at work. However, a minority may be exposed to concentrations which affect health. Policies to mitigate this exposure should be considered, such as the use of respiratory protective equipment, improved ventilation during visits, and interventions to reduce smoking in homes.

**‘What’s Important About This Paper?**

While most workers in Scotland are protected from second-hand smoke (SHS) at work, home care workers (HCWs) are now. This study found that HCWs are exposed to harmful levels of SHS in 21% of home visit, and are concerned about this exposure. Changes to policy should be considered by organisations providing home care to reduce harmful exposure.

## Introduction

Breathing second-hand tobacco smoke (SHS) at work is known to increase the risks of cancer as well as respiratory and cardiovascular conditions including exacerbations of asthma, heart attacks and strokes (Howard, 2004). Since 2006/7 the UK has reduced the number of workers exposed to SHS significantly. At a population level, data from Scotland indicates the proportion of non-smoking adults who show biological evidence of exposure to SHS has fallen from 83 to 19%, with most of this reduction the result of legal restrictions in workplaces and social settings (Semple *et al.*, 2018). With smoke-free prisons introduced in 2018 further protecting approximately 50,000 UK prison staff from exposure to SHS (Demou *et al.*, 2020), there are now few worker groups that continue to be exposed to this known hazard.

Domiciliary health and care workers, also known as home care workers (HCWs), are a large, and growing group of over 600 000 workers (UK Home Care Association, 2016) in the UK who perform day-to-day work tasks that involve spending time in patients’ homes where smoke-free regulations do not apply. Thus, they are one of the final occupational groups likely to experience frequent and high exposure to SHS as part of their work (Dobson *et al.*, 2021).

Domiciliary workers who enter homes where smoking takes place may face similar concentrations of SHS to those experienced by non-smokers who live with a partner or spouse who smokes. Living with a smoker is generally estimated to increase the risk of common illnesses/events such as heart attack, stroke, and lung cancer by about 20–30% (Jamrozik, 2005). Current guidance and policy measures used by Health Boards and Social Care providers to assess and manage the risks to health from SHS are fragmented and often poorly understood. Evidence suggests that SHS exposure is a real concern for many domiciliary workers (Angus and Semple, 2019) who feel left behind in terms of exposure to SHS when almost all other workers are protected through legislation.

Policies and guidance to manage domiciliary workers’ exposure to SHS are out-dated (Royal College of Nursing, 2006) and anecdotal evidence suggests they are rarely implemented. For example, the Royal College of Nursing advice issued in 2006 advises those receiving care at home not to smoke for 1 h prior to a visit but this is at odds with more recent public health messaging about how long SHS remains in household air, lingering up to 5 h (Semple and Latif, 2014).

A recent review (Angus and Semple, 2019) of the scientific literature has identified poor management of this issue and a need for development in policy and practice to balance the needs and responsibilities of those requiring care in their home with protection for those whose jobs involve providing assistance in domestic settings. Implementing appropriate and proportionate measures to protect HCWs from the harms posed by SHS should be a priority to help protect the health of this often over-looked occupational group.

In this study, we aimed to assess the extent of exposure to SHS among HCWs in Lanarkshire, Scotland. We conducted anonymous self-report surveys with National Health Service (NHS) workers, local government-employed HCWs, and HCWs employed through a private company to ask about perceptions of exposure to SHS. To provide objective measurement, we conducted personal exposure monitoring (PEM) across a sample of NHS and local government HCWs.

## Methods

### Ethical approval

Ethical approval for the study was received from the NHS Cambridge East research ethics committee (20/EE/0121).

### Survey

Surveys were conducted with all three workforce groups as part of existing “Healthy Working Lives” surveys coordinated by NHS Lanarkshire. These surveys were delivered electronically to all HCWs within each organisation in November 2019 (NHS Lanarkshire), October 2020 (North and South Lanarkshire Councils) and April 2021 (the private company, unnamed here for confidentiality). Due to the means of delivery (internally by organisation) we did not receive information on the total number of recipients, and cannot estimate a response rate. Every survey question was optional, so the number of responses per question may be lower than the total number of survey responses.

### Personal exposure monitoring

PEM consisted of four triangulating measures: PM_2.5_ monitoring, air nicotine sampling, salivary cotinine measurement, and participant diaries.

Fine particulate matter (PM_2.5_), a commonly-used marker for the presence of SHS in indoor environments (Apelberg *et al.*, 2013), was measured in real-time using the PurpleAir PA-II-SD optical particle counter (PurpleAir Inc, Draper, USA). This monitor has been tested extensively for use indoors and outdoors, using the well-characterised Plantower PMS5003 sensor (Beijing Plantower Co., Beijing, PRC) to estimate mass concentrations of PM_2.5_. PurpleAir monitors were attached to the outside of weighted backpacks and powered using an external USB battery inside the bag. These devices were switched on and logged data from the beginning of the worker’s shift through to collection at the end of the shift. They were carried throughout the course of the working day. On entering a patient home, participants were instructed to place the bag on an elevated surface (such as a table or chair) within the room where they were providing care.

To understand the contribution of ambient PM_2.5_ to indoor air during our monitoring periods, we downloaded hourly PM_2.5_ data from the Scottish Air Quality Network’s Fidas PM monitor (Palas GmbH, Karlsruhe, Germany) in Hamilton (Ricardo Energy and Environment, 2019). This roadside monitor is located in the centre of Hamilton, an urban town which is the administrative seat of South Lanarkshire and close to most PEM sites. For comparison, mean hourly concentrations were calculated for each partial hour of PEM. Natural logs of both concentrations were taken (to ensure a normal distribution), infinite values discarded, and the two datasets compared in a linear model.

Air nicotine samplers were used to estimate direct contamination with SHS (Apelberg *et al.*, 2013). These samplers comprised sodium bisulphate filters within a plastic housing, with a semi-permeable membrane allowing vapour-phase nicotine to pass through and settle on the filter. These samplers were clipped to the outside of each participant’s clothing, either at lapel height (where possible) a pocket on the shirt or trousers. Participants were instructed to ensure that the samplers remained open to the air even where personal protective equipment (PPE) was worn, moving the sampler if necessary. Air nicotine samplers were provided and analysed by the Particulate Matter Research Centre, Johns Hopkins University (Baltimore, USA), using previously published methods (Cardozo *et al.*, 2019).

Salivary cotinine was analysed from saliva samples taken at the start and end of each participant’s shift. Saliva samples were extracted using salivettes (Sarstedt Inc., Nümbrecht, Germany) containing cotton swabs. Participants were asked not to eat or drink for 30 min before sampling and to hold the cotton swab in their mouths for 5 min during the sample period. ABS Labs (York, UK) provided analytical services.

Finally, shift diaries were completed to assess self-reported exposure to SHS, including smell of SHS, as well as times spent making home visits. These diaries also included information on smoking and vaping by participants.

### PEM analysis

We combined diary data with direct reading PM_2.5_ data to calculate concentrations during home visits. PM_2.5_ data taken during home visits as indicated in participant shift diaries (± 15 min to account for estimated and rounded visit times) were extracted from each measurement and mean concentrations of PM_2.5_ were calculated for those times.

Descriptive statistics (including medians and quartiles) were calculated for all home visits and by workforce type, and workforce concentrations were compared through the use of Student’s *t*-test on log-transformed mean shift concentrations. Salivary cotinine concentrations below the limit of detection (LOD) were treated as LOD (0.1 ng ml^‐1^) divided by √2. Our analysis intention was to compare post-shift with pre-shift salivary cotinine in non-smoking HCWs and use previously published ‘Rosetta Stone’ equations linking nicotine intake with a SHS-PM_2.5_ equivalent concentration over the shift (Repace *et al.*, 2006). From the Purple Air data we calculated time-weighted averages of PM_2.5_ concentrations for each participant over their shift.

To assess the potential contribution of SHS to workers’ risk of experiencing ill-health associated with SHS exposure, we analysed the reasonable worst-case scenario from our PEM data on PM_2.5_. We defined this as the 75th percentile of both number of visits and visit length and the 95th percentile of mean PM_2_.5 concentration over a visit. We calculated a time-weighted average of this scenario and compared it to our estimated value for ambient PM_2.5_, multiplied by a standard indoor:outdoor ratio (0.6).

## Results

### Surveys

A total of 490 survey responses were received—95 from NHS Lanarkshire, 64 from the private company, 84 from North Lanarkshire Council, 311 from South Lanarkshire Council (representing 9% and 25% of home care staff at each council respectively). As the survey was included in pre-existing Healthy Working Lives surveys and sent to a wide group of staff, it was not possible to estimate response rates for the NHS or private company. 473 (89%) of 530 respondents were women, while 55 (10%) were men, and none reported other gender identities.

72% of respondents considered themselves to be exposed to SHS as part of their work, but this was highly dependent on their workplace—98% of North Lanarkshire Council HCWs, 80% of South Lanarkshire Council HCWs, and 85% of private company HCWs considered themselves exposed to SHS, but only 19% of NHS Lanarkshire HCWs did (Table 1).

**Table 1.** Survey responses to questions on SHS exposure at work (by workplace).

“Are you exposed to second-hand tobacco smoke as part of your work?”	Yes	No		Total	
NHS Lanarkshire	15 (18%)	80 (94%)		85	
North Lanarkshire Council	82 (98%)	2 (2%)		84	
South Lanarkshire Council	245 (80%)	61 (20%)		306	
Private company	53 (85%)	9 (15%)		62	
Total	395 (74%)	152 (28%)		537	
“How often are you exposed to second-hand smoke in the following places … patients in their homes?”	Daily	At least weekly	Less often than once per week	Never	Total
NHS Lanarkshire	3 (20%)	1 (7%)	3 (20%)	8 (53%)	15
North Lanarkshire Council	51 (62%)	25 (30%)	6 (7%)	0 (0%)	82
South Lanarkshire Council	111 (47%)	83 (35%)	38 (16%)	4 (2%)	236
Private company	26 (53%)	17 (35%)	5 (10%)	1 (2%)	49
Total	191 (50%)	126 (33%)	52 (14%)	13 (3%)	382

Around 50% of those HCWs reported that they were exposed to SHS in patients’ homes on a daily basis, while a further 33% reported that they were exposed at least weekly (Table 1).

### PEM recruitment and participant information

PEM took place during October–December 2020 and May–August 2021. We recruited a total of 32 PEM participants, 23 from the NHS, and nine from South Lanarkshire Council. 7 participants were smokers while 25 were non-smokers.

### Data integrity

29 of the 32 recruits provided full PM_2.5_ data, with monitor failures leading to total data loss for three. 31 participants provided a total of 63 saliva samples which were analysed for salivary cotinine, however nine samples (from five participants) were not analysed due to insufficient volume.

### Diaries

Diary analysis showed that the collected PM_2.5_ data covered 82 separate home visits. Home visits lasted for a median of 20 min (IQR 15–30) and the median number of visits made by a participant over their shift was three (IQR 2–5).

### PM_2.5_ exposure during home visits

Mean PM_2.5_ concentrations were calculated for each home visit. The median of these concentrations across the 82 monitored home visits was 4 µg m^‐3^ (IQR 2–17). Mean PM_2.5_ exceeded the World Health Organisation’s 2010 guideline limit of 25 µg m^‐3^ for 24 h exposure (World Health Organization, 2010) during 17/82 (21%) of home visits, and exceeded the US Environmental Protection Agency’s “unhealthy” value of 55 µg m^‐3^ during 12/82 (15%) of visits (Environmental Protection Agency, 2013). The maximum household visit concentration detected was 225 µg m^‐3^.

**Figure 1.**
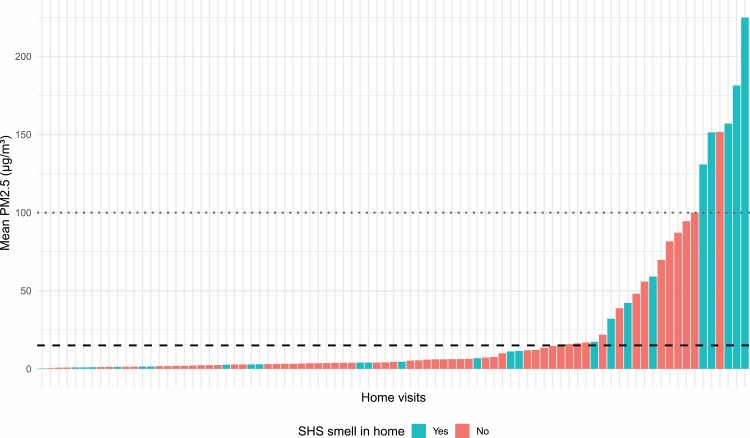
Mean PM2.5 concentration during each monitored home visit, coloured by participant’s report of SHS odour. Dashed line represents WHO pre-2021 guideline limit (25 µg m^‐3^) while the dotted line represents US EPA “unhealthy” level (55 µg m^‐3^).

Participants were asked to note if SHS odour was detected during a home visit. The median concentration during the 25 home visits where the smell of SHS was reported was 4.5 µg m^‐3^ (IQR 1–42) and was 4.1 µg m^‐3^ (IQR 2.8–13.5) in the 57 homes where the worker did not note an odour of SHS. Data from each home visit is given in Fig. 1.

### PM_2.5_ exposure over full shifts

**Figure 2.**
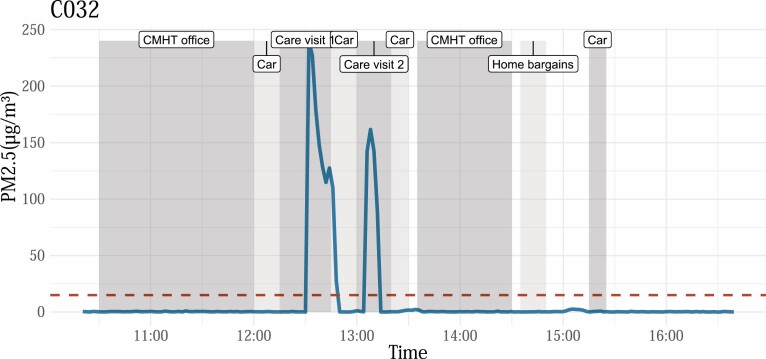
An example of whole-shift monitoring for participant C007, showing activity periods (from the participant’s diary) and real-time PM2.5 concentrations as measured by the PurpleAir monitor. Periods of elevated concentration are visible during home visits 3, 4 and 5. Dashed line represents WHO pre-2021 guideline limit (25 µg m^‐3^).

The median full shift mean PM_2_.5 concentration detected was 6 µg m^‐3^ (IQR 2–14). A representative example of whole-shift monitoring is given in Fig. 2.

### Ambient PM_2.5_

Hourly mean PM_2_.5 concentrations in Hamilton were low during PEM periods (median 3.7 µg m^‐3^, IQR 2.8–4.4, maximum value recorded 10.9). When natural logarithms of indoor and outdoor hourly concentrations were compared in a linear model, *R*^2^ was 0.06, indicating a weak link between the values.

### Clinical significance of SHS-related PM_2.5_ exposure

We calculated a time-weighted average PM_2.5_ concentration for the reasonable worst-case scenario worker, and compared this to a value based solely on ambient air pollution. To do this, we used the following values: the 75th percentile for visits per day (5), the 75th percentile for length of home visit (30 min), the 95th percentile for mean PM_2_.5 concentration measured during a home visit (147 µg m^‐3^), and the median ambient PM_2.5_ during home visits (3.7 µg m^‐3^) multiplied by 0.6, a standard value to estimate the indoor:outdoor ratio of PM_2.5_ concentrations in the UK (leading to the value 2.2 µg m^‐3^).

We calculated that a worker in this reasonable worst-case scenario would be exposed to a mean PM_2_.5 concentration of 17.3 µg m^‐3^ over the course of a 24 h day, compared with a worker who was not exposed to SHS, who would be exposed to a mean concentration of 2.2 µg m^‐3^ in a work day—an increase of 15.1 µg m^‐3^.

### Air nicotine

Due to problems related to international shipping, 16 samples were not delivered to Johns Hopkins University and could not be analysed.

None of the 17 analysed air nicotine samples were above LOD [0.033 μg m^‐3^ at an effective sampling rate of 25 ml min^‐1^ (Cardozo *et al.*, 2019)]. This is consistent with the relatively low levels of SHS-related PM_2.5_ detected in most homes and the short duration of monitoring (a maximum of 6 h, 36 min).

### Salivary cotinine

Eight participants were excluded from this analysis as they reported that they smoked. Of the 25 non-smoking individuals in this study, 24 provided both pre- and post-shift saliva samples. Four participants provided at least one sample with insufficient volume for analysis, so 20 samples were analysed.

Six participants had pre-shift values above LOD (range 0.175–620 ng ml^‐1^), while nine had post-shift values above LOD (range 0.1–564 ng ml^‐1^). The median change from pre- to post-shift was 0 ng ml^‐1^ (IQR 0–0.16).

## Discussion

Many HCWs in Lanarkshire consider themselves to be exposed to SHS during home care visits. Our PEM results suggest that HCWs are exposed to SHS during about 1 in 5 of these visits, and in these cases may be exposed to variable concentrations of PM_2.5_. The highest concentrations measured during some visits exceeded 100 µg m^‐3^ with 15% of visits involving an in-home PM_2.5_ concentration exceeding the US EPA ‘unhealthy’ cut-point of 55 µg m^‐3^ (over a 24 h duration).

We believe that HCWs in Lanarkshire are likely to be broadly representative of HCWs across Scotland. Lanarkshire is a largely urban area in the central belt of Scotland, the area where the majority of the population lives. While much of the area is relatively deprived (with North and South Lanarkshire ranking 6th and 10th of 32 council areas respectively for percentage of the population in the highest quintile of deprivation), the form of work is unlikely to change based on this area.

There was a large difference in self-reported exposure between NHS HCWs and HCWs in other workplaces (19% in the NHS vs 80%+ in council and private workplaces). This may relate to differences in the duties performed by workers in each workplace—NHS workers may be more specialised and conduct fewer home visits than others, with consequently fewer opportunities for SHS exposure. During the study, HCWs and others in NHS Lanarkshire reported that the number and duration of home visits had been minimised due to the COVID-19 pandemic. This may have reduced exposure compared to council and private HCWs, who may have been providing regular care rather than appointments scheduled for specific healthcare needs.

While HCWs are likely exposed to a clinically insignificant amount of PM_2.5_ derived from SHS exposure for much of their time, a worker in a reasonable worst-case scenario (Marquart *et al.*, 2001) may be exposed to approximately 15 µg m^‐3^ of additional PM_2.5_ over the course of a day compared to a similar colleague not involved in visits to homes where smoking takes place. Increased exposure to PM_2.5_ of more than 10 µg m^‐3^ over the course of a year have been found to lead to significant increases in both cardiopulmonary (23%) and all-cause mortality (12%) (Pope *et al.*, 2017). It is worth noting that these figures are at a population level and HCWs are likely to be generally fit, healthy, working adults who may be at lower risk of illness from such exposure.

While the majority of HCWs in this study, and even the reasonable worst-case example generated from our data experience a relatively small increase in their overall 24 h PM_2.5_ exposure from SHS, it should be noted that very few non-home workers in the UK are now expected to work in environments where they are significantly exposed to SHS (Dobson *et al.*, 2021). With recent restrictions on smoking inside prisons (Demou *et al.*, 2020), homes are now the last remaining indoor environment where SHS exposure occurs at work. It is also worth noting that the WHO state that there is ‘no safe level of exposure to SHS’ (NHS Inform 2014) and that short peaks of PM_2.5_ exposure such as those measured in this study may also be associated with acute health effects such as triggering exacerbations of asthma and COPD.

Mitigation measures could potentially reduce HCW’s exposure to SHS. For instance, updating guidance around smoking indoors before a care visit to highlight the potential 5-h linger time used in the Scottish Government’s Take It Right Outside campaign (Cheek *et al.*, 2021) could result in clients leaving more time between smoking indoors and receiving a home care visit. Other potential mitigation measures could include the use of fitted N95 or similar masks to reduce workers’ exposure to SHS, or potentially the use of air cleaning devices (Rice *et al.*, 2018) in homes to reduce household PM_2.5_ concentrations. Future research could address these possibilities with HCWs and clients, exploring how to balance the needs of smokers requiring care in their homes and the rights of workers to a safe working environment.

### Limitations

We cannot be certain that PM_2.5_ detected during home visits represents smoking behaviour, due to the lack of data on airborne nicotine. However, there are few other potential sources of indoor PM_2.5_ of the magnitude detected in this study, and high levels of PM_2.5_ were correlated with SHS odour. PM_2.5_ is a well-recognised and extensively employed marker of SHS concentrations in indoor environments where people are known to smoke (Apelberg *et al.*, 2013).

In common with other studies conducted during the early stage of the COVID-19 pandemic, both recruitment and research were challenging. We conducted the PEM part of this research over two periods (October–December 2020 and May–August 2021), controlled by legal restrictions on gathering and working in Scotland as well as the internal regulations of participating organisations. Care providers were severely impacted by the pandemic, citing a heavily increased workload, and workers from the NHS and from South Lanarkshire reported changes to working patterns driven by the changing situation (with reduced staff coverage and fewer home visits common responses). Due to these workload pressures, staff from the private company and from North Lanarkshire council could not be recruited to PEM research. Consequently, our PEM sample was smaller than we had intended at the outset of this work.

## Conclusions

SHS remains a hazard at work for HCWs. While most home visits in the UK do not involve SHS exposure, a substantial fraction involve potentially harmful concentrations of SHS-related PM_2.5_, and the most severely impacted workers may have increased risk of acute and chronic illness as a result. Consideration should be given to potential mitigation measures to reduce this risk.

## Data Availability

The data underlying this article will be shared on reasonable request to the corresponding author.
